# Impact of an Interleukin-1 Receptor Antagonist and Erythropoietin on Experimental Myocardial Ischemia/Reperfusion Injury

**DOI:** 10.1100/2012/737585

**Published:** 2012-05-02

**Authors:** Christina Grothusen, Angelika Hagemann, Tim Attmann, Jan Braesen, Ole Broch, Jochen Cremer, Jan Schoettler

**Affiliations:** ^1^Department of Cardiovascular Surgery, University Medical Center of Schleswig-Holstein, Campus Kiel, Arnold-Heller-Straße 3, Haus 18, 24105 Kiel, Germany; ^2^Department of Pathology, University Medical Center of Schleswig-Holstein, 24105 Kiel, Germany; ^3^Department of Anaesthesiology and Intensive Care Medicine, University Medical Center of Schleswig-Holstein, 24105 Kiel, Germany

## Abstract

*Background*. Revascularization of infarcted myocardium results in release of inflammatory cytokines mediating myocardial reperfusion injury and heart failure. Blockage of inflammatory pathways dampens myocardial injury and reduces infarct size. We compared the impact of the interleukin-1 receptor antagonist Anakinra and erythropoietin on myocardial ischemia/reperfusion injury. In contrast to others, we hypothesized that drug administration prior to reperfusion reduces myocardial damage. *Methods and Results*. 12–15 week-old Lewis rats were subjected to myocardial ischemia by a 1 hr occlusion of the left anterior descending coronary artery. After 15 min of ischemia, a single shot of Anakinra (2 mg/kg body weight (bw)) or erythropoietin (5000 IE/kg bw) was administered intravenously. In contrast to erythropoietin, Anakinra decreased infarct size (*P* < 0.05, *N* = 4/group) and troponin T levels (*P* < 0.05, *N* = 4/group). *Conclusion*. One-time intravenous administration of Anakinra prior to myocardial reperfusion reduces infarct size in experimental ischemia/reperfusion injury. Thus, Anakinra may represent a treatment option in myocardial infarction prior to revascularization.

## 1. Introduction

Acute total occlusion of a coronary artery is regarded as the underlying cause of acute myocardial infarction (AMI), one of the most common causes of sudden cardiac death in the western world [1, 2]. Several large-scale clinical trials have demonstrated the importance of early reperfusion strategies to improve the extent of myocardial damage as well as patient outcome [3]. So far, current guidelines recommend the interventional treatment via percutaneous transluminal coronary angioplasty (PTCA) of the culprit coronary lesion followed by stent implantation in the setting of AMI, while operative myocardial revascularization—if needed—should generally be performed several days later [4, 5]. However, randomized clinical trials concerning this topic are missing. Arguments against coronary artery bypass surgery (CABG) in AMI include the idea of an overwhelming reperfusion injury caused by prolonged ischemia that may negatively influence the benefit of operative myocardial revascularization [6]. In addition, CABG in AMI may include the enhanced risk for perioperative complications and worsening of myocardial inflammation by exposing the patient to extracorporeal circulation. Various studies have proven that myocardial reperfusion itself results in an inflammatory response that ultimately leads to cell death and possible loss of cardiac function. The underlying mechanisms include the interplay of a multitude of inflammatory mediators [7]. In this context, Interleukin (IL)-1*α* and *β* via their receptor IL-1 type I and activation of nuclear factor (NF)*κ*B act as classical inflammatory cytokines, mediating leukocyte chemotaxis, macrophage activation, reactive oxygen species formation, endothelial dysfunction, and cardiomyocyte apoptosis [8, 9]. Thus, inhibition of IL-1 receptor activation has been recognized as an interesting anti-inflammatory target. For example, enhanced levels of intrinsic IL-1 receptor antagonist (IL1-Ra) can be found during acute myocardial infarction, which correlate with the extent of infarct size [10, 11]. Anakinra is a nonglycosylated, recombinant human, competitive inhibitor of IL-1*α* and *β* signaling through binding to the IL-1 receptor. Anakinra has already been demonstrated to possess cardioprotective properties in different experimental and clinical settings [12, 13]. While experimental evidence for a protective role of erythropoietin in myocardial ischemia has been promising, clinical trials so far have not been able to proof this hypothesis. In particular, erythropoietin dosage, way of application and treatment duration, may critically influence study outcome [14]. In contrast to other studies, we here hypothesized, that application of anti-inflammatory drugs like Anakinra and erythropoietin already prior to myocardial reperfusion might positively influence reperfusion damage and, thereby, may qualify these drugs for consideration as preclinical treatment options in the setting of AMI.

## 2. Material and Methods

### 2.1. Animals

12–15-week old male Lewis rats (Charles River, Sulzfeld, Germany) weighing 280–350 g were used for ischemia/reperfusion experiments. All animals were kept according to the Institutional guide for the care and use of laboratory animals. All procedures were approved by the Institution's facility for Laboratory Animal Science.

### 2.2. Myocardial Ischemia/Reperfusion Injury

Rats were initially narcotized by inhalation of ether followed by a subcutaneous injection of  20% urethane (0.75 mL/100 g) and tracheal intubation. Maintenance of anesthesia was achieved by inhalation of isoflurane (0.5–1.5% isoflurane/100% oxygen). The left femoral vein was cannulated for drug administration. After lateral thoracotomy and opening of the pericardial sack, the left anterior descending artery (LAD) was exposed and occluded by ligation using 5–0 Prolene suture (Johnson&Johnson, Ethicon Biosurgery, USA). Animals were randomly assigned to 4 experimental groups (*N* = 4 animals/group). Group 1 (control) received a bolus of physiological saline solution 15 minutes (min) after onset of myocardial ischemia. Ischemia was maintained for 1 hour (hr) followed by 3 hrs of reperfusion. Group 2 (Anakinra) received 2 mg/kg body weight (bw) Anakinra (Kineret, Amgen GmbH, Germany) 15 min after onset of ischemia. Group 3 (erythropoietin) was treated with 5000 IE/kg bw erythropoietin (Neorecormon, Hoffmann-La Roche Ltd., Germany) 15 min after onset of ischemia. Both, group 2 and 3 underwent 1 hr of myocardial ischemia followed by 3 hrs of reperfusion after which rats were sacrificed for further analyses.

### 2.3. Assessment of Infarct Size and Area at Risk

After 3 hrs of reperfusion, the LAD was reoccluded, and stainings were performed. In short, Evans blue dye (1%, 3–5 mL, Sigma-Aldrich, Germany) was injected into the beating right ventricular cavity to distinguish between ischemic (area at risk) and nonischemic myocardium. To determine the infarct size, the heart was sliced into five 2 mm thick sections, each was weighed and incubated with a 1.5% (W/V) triphenyltetrazolium chloride (TTC) solution for 30 min at 37°C followed by immersion in liquid-nitrogen frozen 2-methylbutane solution for 10 min. Sections were cryo-sliced into 60 *μ*m slides. Photographs were taken, and the ischemic area at risk (unstained by Evans blue dye) and the infarcted area (unstained by TTC) were measured in a blinded fashion using the axio vision 3.1 software (Zeiss, Oberkochem, Germany). In addition, infarct size and area at risk were weighed.

### 2.4. Systemic Inflammatory Cytokine Levels

To investigate the possible impact of the different treatment regimens on circulating cytokine levels, sera of 2 animals per group were pooled. Overall, 4 samples per group were subjected to ELISA (RayBio Rat Cytokine Antibody Array, RayBiotech, Norcross, USA) following the manufacturer's protocol.

### 2.5. Troponin T Levels

Troponin T (TnT) levels were determined as indicators of myocardial injury. Blood samples were collected before the sacrifice of animals, centrifuged, and the serum was frozen at −20°C. Serum TnT was measured using commercial kits (Roche Diagnostics, Basel, Switzerland) following the manufacturer's instructions.

### 2.6. Statistical Analysis

 All results are expressed as mean ± SEM. Groups were compared using 1-way ANOVA. Variables within a group were compared using a paired *t*-test. *P* values ≤ 0.05 were considered significant.

## 3. Results

### 3.1. Anakinra Applied Prior to Myocardial Reperfusion but Not Erythropoietin Reduces Infarct Size

**Figure 1:**
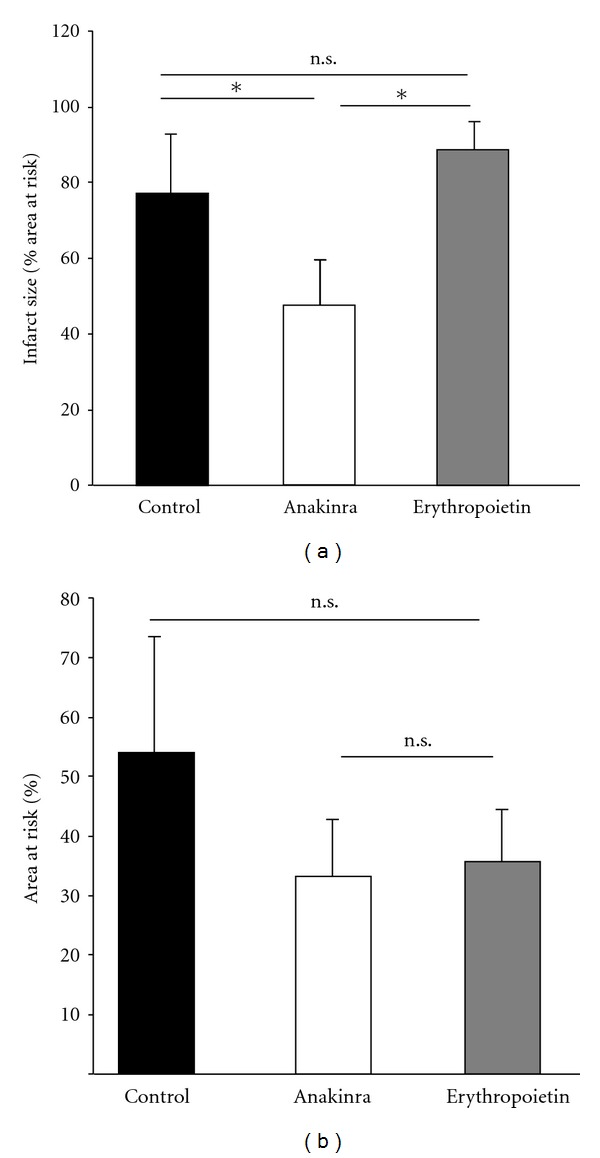
(a) Impact of Anakinra and erythropoietin on infarct size. One-time intravenous administration of Anakinra prior to reperfusion resulted in a significant reduction of infarct size expressed as infarct mass in relation to area at risk mass (%) compared to controls or animals receiving erythropoietin (*P < 0.05, *N* = 4/group). (b) Impact of Anakinra and erythropoietin on area at risk. One-time intravenous administration of Anakinra or erythropoietin prior to reperfusion did not significantly influence area at risk (n.s. = non significant, *N* = 4/group).

One-time intravenous administration of 2 mg/kg bw Anakinra prior to myocardial reperfusion significantly reduced infarct size (expressed as infarct mass in relation to area at risk mass) compared to animals that received erythropoietin or saline solution (47.6 ± 6.0% versus 76.2 ± 12.9% and 77.1 ± 7.8%, *N* = 4 animals/group, *P* < 0.05, Figure 1(a)). Area at risk did not differ between the groups (Figure 1(b)).

### 3.2. Anakinra Applied Prior to Myocardial Reperfusion but Not Erythropoietin Reduces Troponin T Levels

**Figure 2:**
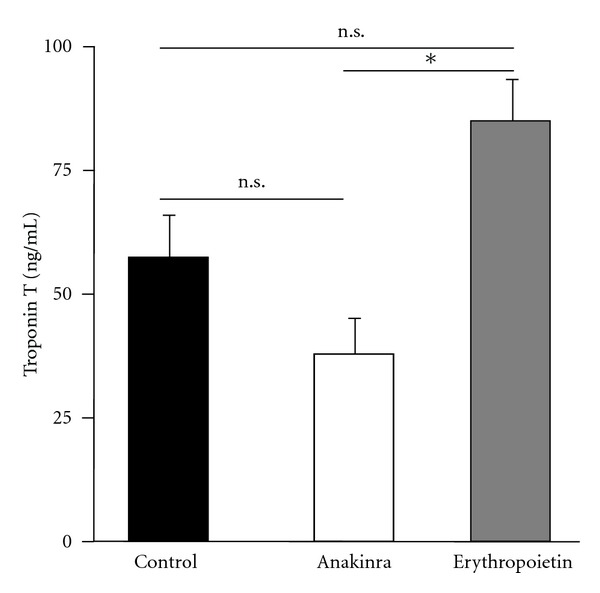
Impact of Anakinra and erythropoietin on troponin T levels. One-time intravenous administration of Anakinra prior to reperfusion resulted in a significant reduction of troponin T levels compared to animals receiving erythropoietin (*P* < 0.05, *N* = 4/group), whereas no significant difference compared to rats receiving saline solution (control) was found.

Troponin T (TnT) levels, which have been demonstrated to correlate with infarct size in rats, were significantly lower in Anakinra-treated animals compared to rats receiving erythropoietin (40.4 ng/mL versus 57.8 ng/mL, *N* = 4/group, *P* < 0.05, Figure 2). However, no significant difference between Anakinra-treated animals and untreated animals was observed. Levels of creatinkinase (CK) or CK-MB did not differ between the groups (data not shown) [15].

### 3.3. Anakinra and Erythropoietin Applied Prior to Myocardial Reperfusion Do Not Influence Systemic Inflammatory Cytokine Levels

**Figure 3:**
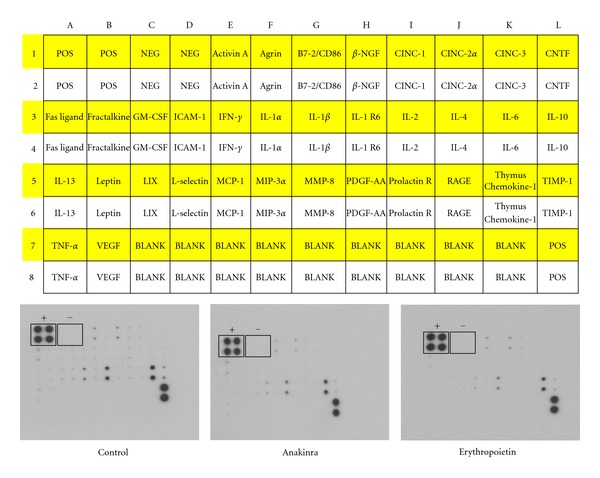
Impact of Anakinra and erythropoietin on circulating inflammatory cytokines. Representative pictures demonstrating no differences in the relative expression levels of inflammatory cytokines depicted as black spots (*N* = 4 samples/group, + indicating positive control spots, − indicating negative control spots). Cytokine panel (top) published with kind permission of RayBiotech, Inc.

To investigate whether modulation of inflammatory signaling pathways by application of Anakinra or erythropoietin may influence systemic cytokine levels, sera of animals were subjected to ELISA (*N* = 4 samples/group). However, we did not find any differences in cytokine levels between the groups (Figure 3). 

## 4. Discussion

In contrast to previous studies, we here hypothesized that a one-time intravenous administration of the IL-1 receptor antagonist Anakinra or erythropoietin already prior to reperfusion may limit myocardial I/R injury. We report that Anakinra, but not erythropoietin, reduces infarct size in this experimental setting. Myocardial reperfusion has been demonstrated to cause cardiomyocyte death, microvascular dysfunction, ventricular arrhythmias, and, ultimately, loss of cardiac function resulting in heart failure [7]. During coronary artery occlusion, ischemia in the dependent tissue results in change of cellular metabolism from aerobic to anaerobic energy utilization. Rapid reperfusion, however, may induce an uncontrolled formation of reactive oxygen species that not only serve as chemoattractant for inflammatory cells, but may directly damage cell compartments, such as mitochondria and the sarcoplasmic reticulum [16]. The ischemia-triggered rise in intracellular calcium load is further worsened by reperfusion and may drive cell hypercontraction. The influx of inflammatory cells into the area of former ischemia contributes to all of these mechanisms. Therefore, modulating the inflammatory reaction during myocardial reperfusion has been an attractive target for experimental as well as clinical trials. IL-1*α* and *β* via activation of the IL-1 type I receptor have been indicated to promote a multitude of inflammatory processes. In addition, both cytokines have been implicated in cardiac remodeling and heart failure. Thus, blocking the IL-1 type I receptor by Anakinra seems a promising therapeutic target. A cardioprotective effect of Anakinra has already been proposed by the results of different experimental studies. For example, cardiac overexpression of IL-1 receptor antagonist reduced infarct size in a rat model of myocardial infarction. The underlying mechanisms may involve a decrease in cardiomyocyte apoptosis, as indicated by a study of Abbate and colleagues [17]. In addition, Anakinra positively influences endothelial dysfunction, reduced oxidative stress, and improved ventricular function in patients with rheumatoid arthritis, a known risk factor for cardiovascular events [18]. As indicated by the findings of many other groups in experimental and clinical trials, time, dosage, duration, and way of drug application may lead to essentially different results regarding myocardial reperfusion injury. Based on these facts and in contrast to other groups, we hypothesized that the extent of reperfusion injury may be most efficiently influenced by administration of the anti-inflammatory drug already prior to reperfusion. Thereby, the experimental design of this study aimed at imitating not only the possibility of a more specific prehospital treatment of acute myocardial ischemia. Instead, we also speculated that an anti-inflammatory drug administration prior to reperfusion may improve the management of patients undergoing coronary artery bypass surgery, as myocardial ischemia and reperfusion play an important role in this setting [19]. In this context, we also decided to apply Anakinra and erythropoietin via a central venous access site in order to quickly achieve systemic drug circulation despite circulatory depression during cardiac ischemia. To our knowledge, this approach has not been reported by other groups so far. As stated above, we did not find any effect of erythropoietin on myocardial infarct size in the study presented. Erythropoietin is a hematopoietic hormone primarily produced in the kidney in response to hypoxia. However, beside its impact on hematopoiesis, erythropoietin also modulates cardiovascular cell function. In context with myocardial ischemia, erythropoietin has been shown to decrease cardiomyocyte apoptosis in different experimental settings [20, 21]. In addition, erythropoietin seemingly decreases the inflammatory response during myocardial reperfusion by modulating nitric oxide release [22, 23]. However, onset and duration of treatment as well as dosage may again be crucial for the extent and the kind of biological impact achieved by erythropoietin. So far, clinical trials involving erythropoietin treatment in acute myocardial infarction in patients have failed to prove any benefit [24]. Thus, further studies are needed to clarify the role of this hormone in myocardial ischemia and reperfusion. Neither erythropoietin nor Anakinra modulated the levels of circulating inflammatory cytokines in the study presented. We speculate that this observation may either be due to the circumstance that systemic cytokine levels may not reflect local processes. In addition, the time point of sample extraction may not have been accurate in order to detect a modulation of the systemic inflammatory state. To summarize, one-time application of Anakinra prior to myocardial reperfusion—in contrast to erythropoietin—leads to a decreased extent of experimental myocardial I/R injury and, therefore, may not only be considered as a treatment option after revascularization but instead may also be beneficial in the very early phase of acute myocardial infarction.

## References

[1] Bailey JJ, Berson AS, Handelsman H, Hodges M (2001). Utility of current risk stratification tests for predicting major arrhythmic events after myocardial infarction. *Journal of the American College of Cardiology*.

[2] McGovern PG, Jacobs DR, Shahar E (2001). Trends in acute coronary heart disease mortality, morbidity, and medical care from 1985 through 1997: the minnesota heart survey. *Circulation*.

[3] Simoons ML, Boersma E, Maas AC, Deckers JW (1997). Management of myocardial infarction: the proper priorities. *European Heart Journal*.

[4] Wijns W, Kolh P, Danchin N (2010). Guidelines on myocardial revascularization: the task force on myocardial revascularization of the european society of cardiology (ESC) and the european association for cardio-thoracic surgery (EACTS). *European Heart Journal*.

[5] Weiss ES, Chang DD, Joyce DL, Nwakanma LU, Yuh DD (2008). Optimal timing of coronary artery bypass after acute myocardial infarction: a review of California discharge data. *Journal of Thoracic and Cardiovascular Surgery*.

[6] Morrison DA, Sacks J (2003). Balancing benefit against risk in the choice of therapy for coronary artery disease: lesson from prospective, randomized, clinical trials of percutaneous coronary intervention and coronary artery bypass graft surgery. *Minerva Cardioangiologica*.

[7] Yellon DM, Hausenloy DJ (2007). Myocardial reperfusion injury. *The New England Journal of Medicine*.

[8] Merhi-Soussi F, Kwak BR, Magne D (2005). Interleukin-1 plays a major role in vascular inflammation and atherosclerosis in male apolipoprotein E-knockout mice. *Cardiovascular Research*.

[9] Chamberlain J, Francis S, Brookes Z (2009). Interleukin-1 regulates multiple atherogenic mechanisms in response to fat feeding. *PLoS One*.

[10] Parti G, D’Ambrosio A, Mega S (2004). Early Interleukin-1 receptor antagonist elevation in patients with acute myocardial infarction. *Journal of the American College of Cardiology*.

[11] Patti G, Mega S, Pasceri V (2005). Interleukin-1 receptor antagonist levels correlate with extent of myocardial loss in patients with acute myocardial infarction. *Clinical Cardiology*.

[12] Salloum FN, Chau V, Varma A (2009). Anakinra in experimental acute myocardial infarction-does dosage or duration of treatment matter?. *Cardiovascular Drugs and Therapy*.

[13] Fearon WF, Fearon DT (2008). Inflammation and cardiovascular disease role of the interleukin-1 receptor antagonist. *Circulation*.

[14] Vogiatzi G, Briasoulis A, Tousoulis D, Papageorgiou N, Stefanadis C (2010). Is there a role for erythropoietin in cardiovascular disease?. *Expert Opinion on Biological Therapy*.

[15] O’Brien PJ, Dameron GW, Beck ML (1997). Cardiac troponin T is a sensitive, specific biomarker of cardiac injury in laboratory animals. *Laboratory Animal Science*.

[16] Garcia-Rivas GJ, Torre-Amione G (2009). Abnormal mitochondrial function during ischemia reperfusion provides targets for pharmacological therapy. *Methodist Debakey Cardiovascular Journal*.

[17] Abbate A, Salloum FN, Vecile E (2008). Anakinra, a recombinant human interleukin-1 receptor antagonist, inhibits apoptosis in experimental acute myocardial infarction. *Circulation*.

[18] Ikonomidis I, Lekakis JP, Nikolaou M (2008). Inhibition of interleukin-1 by anakinra improves vascular and left ventricular function in patients with rheumatoid arthritis. *Circulation*.

[19] Sharma M, Ganguly NK, Chaturvedi G, Thingnam SK, Majumdar S, Suri RK (2003). Release of pro-inflammatory mediators during myocardial ischemia/reperfusion in coronary artery bypass graft surgery. *Molecular and Cellular Biochemistry*.

[20] Mastromarino V, Volpe M, Musumeci MB, Autore C, Conti E (2011). Erythropoietin and the heart: facts and perspectives. *Clinical Science*.

[21] Vogiatzi G, Briasoulis A, Tousoulis D, Papageorgiou N, Stefanadis C (2010). Is there a role for erythropoietin in cardiovascular disease?. *Expert Opinion on Biological Therapy*.

[22] Klopsch C, Furlani D, Gabel R (2009). Intracardiac injection of erythropoietin induces stem cell recruitment and improves cardiac functions in a rat myocardial infarction model. *Journal of Cellular and Molecular Medicine*.

[23] Baker JE, Kozik D, Hsu AK, Fu X, Tweddell JS, Gross GJ (2007). Darbepoetin alfa protects the rat heart against infarction: dose-response, phase of action, and mechanisms. *Journal of Cardiovascular Pharmacology*.

[24] Ott I, Schulz S, Mehilli J (2010). Erythropoietin in patients with acute st-segment elevation myocardial infarction undergoing primary: percutaneous coronary intervention a randomized, double-blind trial. *Circulation*.

